# Supplementary material for: Empirical methods for controlling false positives and estimating confidence in ChIP-Seq peaks
Source: BMC Bioinformatics. 2008 Dec 5;9:523. doi: 10.1186/1471-2105-9-523 (PMC2628906; doi:10.1186/1471-2105-9-523)
Supplement: Additional File 1 — A variety of html documents from the USeq web site detailing the available applications, their best usage, output file type descriptions, command line menus, etc. [file 1471-2105-9-523-S1.zip › USeqUserGuides/JavaDoc.zip!/JavaDoc/trans/main/SetNumberIntervalMaker.html]

SetNumberIntervalMaker


|  |  |  |
| --- | --- | --- |
| **Overview** \| **Package** \| **Class** \| **Use** \| **Tree** \| **Deprecated** \| **Index** \| **Help** | |  |
| **PREV CLASS**   **NEXT CLASS** | **FRAMES**    **NO FRAMES**     **All Classes** |  |
| SUMMARY: NESTED \| FIELD \| CONSTR \| METHOD | DETAIL: FIELD \| CONSTR \| METHOD |  |


---


## trans.main Class SetNumberIntervalMaker

```
java.lang.Object
  trans.main.SetNumberIntervalMaker
```

---

``` public class SetNumberIntervalMaker extends java.lang.Object ```

Determines the threshold(s) needed to generate a set number of intervals, multiple score indexes accepted.

---

**Constructor Summary**

`SetNumberIntervalMaker(java.lang.String[] args)`


| **Method Summary** | |
| --- | --- |
| `void` | `addIt()` |
| `Window` | `fetchGoodWindow(double minScoreInt)` |
| `static void` | `main(java.lang.String[] args)` |
| `int` | `makeMockIntervals(double minScoreInt)`             Returns the number of intervals that can be made at a given cutoff |
| `double[]` | `minMaxWindowScores()`             Finds min max |
| `static void` | `printDocs()` |
| `void` | `processArgs(java.lang.String[] args)`             This method will process each argument and assign new varibles |

**Methods inherited from class java.lang.Object**

`equals, getClass, hashCode, notify, notifyAll, toString, wait, wait, wait`

**Constructor Detail**

### SetNumberIntervalMaker

```
public SetNumberIntervalMaker(java.lang.String[] args)
```


**Method Detail**

### minMaxWindowScores

```
public double[] minMaxWindowScores()
```

:   Finds min max

---


### makeMockIntervals

```
public int makeMockIntervals(double minScoreInt)
```

:   Returns the number of intervals that can be made at a given cutoff

---


### addIt

```
public void addIt()
```

---


### fetchGoodWindow

```
public Window fetchGoodWindow(double minScoreInt)
```

---


### processArgs

```
public void processArgs(java.lang.String[] args)
```

:   This method will process each argument and assign new varibles

---


### printDocs

```
public static void printDocs()
```

---


### main

```
public static void main(java.lang.String[] args)
```


---


|  |  |  |
| --- | --- | --- |
| **Overview** \| **Package** \| **Class** \| **Use** \| **Tree** \| **Deprecated** \| **Index** \| **Help** | |  |
| **PREV CLASS**   **NEXT CLASS** | **FRAMES**    **NO FRAMES**     **All Classes** |  |
| SUMMARY: NESTED \| FIELD \| CONSTR \| METHOD | DETAIL: FIELD \| CONSTR \| METHOD |  |


---
